# Regional distribution of body fat in relation to DNA methylation within the *LPL*, *ADIPOQ* and *PPARγ* promoters in subcutaneous adipose tissue

**DOI:** 10.1038/nutd.2015.19

**Published:** 2015-07-06

**Authors:** D Drogan, H Boeing, J Janke, B Schmitt, Y Zhou, J Walter, T Pischon, S Tierling

**Affiliations:** 1 Department of Epidemiology, German Institute of Human Nutrition Potsdam-Rehbruecke (DIfE), Nuthetal, Germany; 2 AOK Research Institute (WIdO), AOK Bundesverband, Berlin, Germany; 3 Molecular Epidemiology Group, Max Delbrueck Center for Molecular Medicine (MDC), Berlin-Buch, Germany; 4 Department of Genetics/Epigenetics, FR8.3 Life Sciences, Saarland University, Saarbrücken, Germany; 5 Institute of Sociology and Demography, University of Rostock, Rostock, Germany

## Abstract

Obesity may be related to differential DNA methylation and thus to differential expression of key genes in adipose tissue metabolism, such as *LPL*, *ADIPOQ* and *PPARγ*. Using subcutaneous adipose tissue (SAT) from 59 individuals of the European Prospective Investigation into Cancer and Nutrition–Potsdam study, we performed quantitative DNA methylation analysis within the promoters of *LPL* (*LPL*-CG1 and -CG2), *ADIPOQ* (*ADIPOQ*-CG1 and-CG2) and *PPARγ* (*PPARγ*-CG1). We then studied DNA methylation in relation to SAT gene expression, body composition measured using whole-body magnetic resonance imaging, body mass index (BMI), waist circumference (WC) and long-term changes in BMI and WC. For *LPL*-CG1 and *LPL-*CG2, higher methylation levels were associated with lower *LPL* expression, but with higher past WC gain. *LPL*-CG1 was also positively associated with BMI, WC, and visceral and subcutaneous fat mass. *ADIPOQ*-CG1 or -CG2 methylation exhibited no association with *ADIPOQ* expression or with anthropometric parameters. *PPARγ*-CG1 methylation was significantly higher in individuals with higher visceral fat mass. Among the investigated sites, *LPL*-CG1 methylation showed the strongest association with gene expression and regional body fat distribution, thereby possibly linking the degree of obesity with major metabolic processes in SAT.

## Introduction

The contribution of epigenetic alterations such as DNA methylation to obesity or obesity-related comorbidities is not completely understood; however, previous studies have linked measures of obesity to differential DNA methylation in blood cells.^1, 2^ Comparable investigations for adipose tissue (AT) are scarce, although AT is the major fat-storage site and releases bioactive compounds that modulate insulin sensitivity and systemic metabolism.^3^

Using human subcutaneous AT (SAT) samples we measured DNA methylation at five CpG positions located within the promoter regions of three genes with perceived impact on metabolic health: (i) *LPL*—encoding lipoprotein lipase that hydrolyses circulating triglyceride-rich lipoproteins and subsequent fatty acid uptake into AT,^4^ (ii) *ADIPOQ*—encoding the insulin-sensitizing hormone adiponectin^5^ and (iii) *PPARγ*—encoding the peroxisome proliferator-activated receptor γ, a transcription factor regulating intermediary metabolism and insulin sensitivity.^6, 7^ These data were used to investigate DNA-methylation levels in relation to SAT expression of these genes, and to body weight, weight gain and body fat distribution.

## Materials and methods

In 2009, a random sample of the European Prospective Investigation (EPIC)–Potsdam cohort was invited for assessing body composition and physical activity. Altogether, 816 participants attended this re-examination and fulfilled the inclusion criteria of having no severe disease within 1 year and no surgery within 3 months before the examination. The study was approved by the Ethics Committee of the medical association of the State of Brandenburg (Germany). All participants provided written informed consent. Our study included 60 participants chosen randomly among participants with available peripheral venous blood and SAT samples (*n*=200). For subsequent statistical analyses, we excluded one participant with missing methylation data.

SAT and visceral AT were measured using whole-body magnetic resonance imaging.^8^ Trained personnel measured waist circumference (WC), body weight and body mass index (BMI). Using past anthropometric data of the EPIC–Potsdam baseline examination (collected on average 14.5 years before our investigation),^9^ we calculated 5-year changes in body weight, BMI and WC.

DNA and RNA were extracted from SAT samples using the Qiagen AllPrep DNA/RNA Mini Kit (Qiagen, Hilden, Germany). Extraction of DNA from blood was performed using the DNeasy Blood & Tissue Kit. Quantity and integrity of purified DNA and RNA was analysed using the NanoDrop Photometer (PeqLab, Erlangen, Germany) and the Bioanalyser (Agilent Technologies, Böblingen, Germany). Two micrograms of RNA were reverse-transcribed to complementary DNA (cDNA) using the High Capacity cDNA Reverse Transcription Kit (Applied Biosystems, Darmstadt, Germany).

Chromosomal DNA (500 ng) was treated with sodium bisulfite using the EZ DNA Methylation kit (Zymo Research, Irvine, CA, USA) according to the manufacturer's protocol. Amplicons were generated using bisulfite-specific primers (Supplementary Table 1). Amplicons were purified using 1 U of ExonucleaseI/Shrimp Alkaline Phosphatase mix at 37 °C for 30 min followed by 80 °C for 15 min. Purified amplicons were subjected to single-nucleotide primer extension with single nucleotide primer extension with ion-pair reversed-phase high performance liquid chromatography (SIRPH) analysis.^10^ Experimental conduction was carried out as described previously^11^ (details are given in Supplementary Table 2). SAT samples from three normal-weight and three obese participants were used (i) for selecting potentially informative CpG positions in the candidate genes using methylation data obtained from Illumina 450K BeadChip arrays and NGS-based bisulfite profiling focusing on absence of sequence polymorphism, high variability of methylation levels and association with BMI/gene expression (date not shown), and (ii) for validating SIRPH-based DNA methylation results with Bi-PROF.^12^ The correlation between both methods was 0.97 (*P*=0.005) for *LPL*-CG2, 0.87 (*P*=0.024) for *ADIPOQ*-CG1 and 0.94 (*P*=0.005) for *PPARγ*-CG1.

SAT gene expression of *LPL*, *ADIPOQ* and *PPARγ* was assessed with real-time PCR using the Applied Biosystems 7500 Fast real-time PCR system with TaqMan technology (ABI, Darmstadt, Germany). The two-step PCR conditions were 20 s at 95 °C, 40 cycles with 3 s at 95 °C and 30 s at 60 °C (5 μl reaction volume, 4 ng template). All samples and controls were run in triplicates. For each amplification cycle, a threshold cycle (*C*_t_) value was obtained, and the Δ*C*_t_ value was calculated as the *C*_t_ difference between target gene and 18S rRNA. Fold upregulation of gene expression compared with weakest expression was calculated using the 2^−ΔΔ*C*t^ method.^13^

Statistical analysis was performed using the SAS Enterprise Guide, release 9.2 (SAS Institute, Cary, NC, USA). We calculated means±s.d. or frequencies of selected participants' characteristics. In linear regression models, we used anthropometric parameters or gene expression as dependent variables, SAT methylation as predictor and age and sex as covariates. The resulting β-coefficients indicate the change in the respective dependent variable associated with a 0.1 unit increase in DNA-methylation levels.

## Results

This analysis included 18 men and 41 women aged 63.1±8.7 years (Table 1).

In SAT probes, *LPL*-CG1 methylation correlated strongly with *LPL*-CG2 methylation (*r*_s_=0.63, *P*<0.001). The correlation between SAT- and blood methylation—available for *LPL* and *ADIPOQ*—was very weak (Supplementary Table 3). *LPL*-CG1 and *LPL*-CG2 methylation levels were inversely related to *LPL* gene expression (Table 2). Each 0.1 unit increase in *LPL*-CG1 methylation was associated with 3.7 kg m^-2^ higher BMI, 1.1 kg higher visceral AT mass and past weight gain of 1.5 kg per 5 years. SAT-methylation levels between *ADIPOQ*-CG1 and *ADIPOQ*-CG2 correlated weakly (*r*_s_=0.29, *P*=0.023), but exhibited no significant association with *ADIPOQ* gene expression or anthropometric parameters (Table 2). With increasing *PPARγ-*CG1 methylation we observed significantly increased body fat and visceral AT mass.

## Discussion

As epigenetic alterations in AT may be associated with obesity-related phenotypes, we performed quantitative analysis of single CpG methylation within the *LPL*, *ADIPOQ* and *PPARγ* promoters. Among the investigated sites, SAT-methylation levels in *LPL*-CG1 exhibited the strongest inverse association with gene expression and the strongest positive association with measures of regional body fat distribution.

According to the ‘AT expandability hypothesis', individuals possess a threshold for preferentially depositing fat in SAT during periods of energy surplus.^14^ Once this threshold is exceeded, fat is also stored in visceral AT, liver or muscles. Given the gatekeeper function of *LPL* to direct fatty acid entry into AT,^4^ *LPL* activity in AT might decrease as a result of chronic energy surplus and limits in lipid-storage capacity. Indeed, we observed that increased fat was associated with increased *LPL* promoter methylation along with decreased *LPL* expression. Although substantially weaker, similar observations were made for *PPARγ*, a key regulator of AT metabolism,^6, 7^ suggesting that the metabolic capacity of SAT is linked with obesity-related epigenetic mechanisms.^15^

Although adiponectin is mainly released by AT, adiponectin levels are decreased in obesity.^5^ One may speculate that reduced transcription due to methylation of *ADIPOQ* sites may contribute to this paradox. Yet, *ADIPOQ-*methylation levels exhibited no association with *ADIPOQ* gene expression or anthropometric parameters in our study, but for future investigations, methylation sites with a stronger association with gene expression are available.^16^

This investigation profits from the availability of human SAT samples and in-depth anthropometric phenotyping of study participants. To our knowledge, no previous human study targeted body fat distribution as well as longitudinal changes in obesity markers in relation to SAT methylation within the *ADIPOQ*, *LPL* or *PPARγ* genes. In view of the limited statistical power, the significant association of *LPL*-CG1 methylation with gene expression and anthropometry is noteworthy. Although we selected three well-established candidate genes of metabolic diseases with exclusive or predominant expression in AT, the focus on only five CpG positions is a limitation. To our knowledge, *ADIPOQ*–CG2 is the only site with comparable data being reported by others—and its SAT-methylation levels were inversely related to *ADIPOQ* expression in females.^17^ As epigenetic marks display cell specificity, any association between DNA methylation and anthropometric markers might have been influenced by changes in cell composition, for example, obesity-induced macrophage infiltration. Although most CpGs of the human genome demonstrate comparable DNA methylation pattern across cell types, homogeneous cell samples are preferable for epigenetic studies.^18^ Furthermore, our data are correlative and we cannot prove the temporal relationship between the investigated variables. Specifically, obesity may be a consequence of DNA methylation established in early development,^19^ but weight change may as well alter DNA methylation during adulthood.^20^ In either case, the regional body fat distribution appears to be associated with differential DNA methylation in genes encoding for key proteins in AT metabolism, thereby possibly linking the degree of obesity with the susceptibility to develop metabolic dysfunction. However, we observed a relation between the methylation status and expression only in one out of five sites.

## Figures and Tables

**Table 1:** Characteristics of 59 randomly selected participants of a sub-study of EPIC–Potsdam

*Characteristics*	*Value*
Age, year, mean±s.d.	63.1±8.7
Male sex, %	30.5
BMI, kg m^−2^, mean±s.d.	28.6±4.6
WC, cm, mean±s.d.	95.5±11.6
SAT, kg, mean±s.d.^a^	20.4±7.4
VAT, kg, mean±s.d.^a^	3.8±1.6
VAT from fat, %, mean±s.d.^a^	16.3±7.4
Total fat from body mass, %, mean±s.d.^a^	30.4±7.1
ΔWeight, kg per 5 years, mean±s.d.^b^	2.1±2.5
ΔBMI, kg m^−2^ per 5 years, mean±s.d.^b^	0.8±1.0
ΔWC, cm per 5 years, mean±s.d.^b^	3.9±2.9
*LPL*-CG1 methylation, mean±s.d.^c^	0.38±0.05
*LPL*-CG2 methylation, mean±s.d.^c^	0.25±0.05
*LPL* gene expression, mean±s.d.^d^	3.09±1.42
*ADIPOQ*-CG1 methylation, mean±s.d.^c^	0.82±0.02
*ADIPPOQ*-CG2 methylation, mean±s.d.^c^	0.56±0.14
*ADIPOQ* gene expression, mean±s.d.^d^	1.92±0.80
*PPARγ*-CG1 methylation, mean±s.d.^c^	0.34±0.08
*PPARγ* gene expression, mean±s.d.^d^	2.24±0.88

Abbreviations: BMI, body mass index; Δ, change; SAT, subcutaneous adipose tissue; VAT, visceral adipose tissue; WC, waist circumference.

a Data derived from whole-body magnetic resonance imaging available for 49 participants.

b Average time between baseline examination (1994–1998) and the current re-examination was 14.5 years.

c Methylation *β*-value measured in DNA from SAT.

d SAT expression data, normalized to the expression of 18S rRNA and relative to the weakest expression observed in this study population.

**Table 2:** Association of *LPL*-, *ADIPOQ*- and *PPARγ*-promoter methylation in DNA from subcutaneous adipose tissue with anthropometric parameters and gene expression

	*LPL-CG1*		*LPL-CG2*		*ADIPOQ-CG1*		*ADIPOQ-CG2*		*PPARγ-CG1*	
	*β*^a^	P*-value*	*β*^a^	P*-value*	*β*^a^	P*-value*	*β*^a^	P*-value*	*β*^a^	P*-value*
BMI (kg m^−2^)	3.7	0.003	1.2	0.330	0.7	0.810	0.1	0.830	0.8	0.350
WC (cm)	9.9	0.001	3.95	0.191	6.0	0.392	1.7	0.131	2.9	0.135
SAT (kg)^b^	6.9	<0.001	2.3	0.347	1.2	0.807	0.7	0.338	2.3	0.091
VAT (kg)^b^	1.1	0.012	1.0	0.060	1.1	0.306	0.2	0.160	0.7	0.013
VAT from fat (%)^b^	0.5	0.730	2.9	0.086	3.9	0.249	0.3	0.544	1.6	0.095
Total fat from body mass (%)^b^	5.7	<0.001	3.4	0.081	0.7	0.859	0.9	0.164	2.3	0.038
ΔWeight (kg per 5 years)^c^	1.5	0.019	0.8	0.217	1.7	0.267	0.3	0.271	0.5	0.205
ΔBMI (kg m^−2^ per 5 years)^c^	0.6	0.011	0.3	0.156	0.6	0.333	0.1	0.281	0.2	0.173
ΔWC (cm per 5 years)^c^	1.4	0.047	1.5	0.033	1.6	0.341	0.4	0.117	0.3	0.467
SAT gene expression^d^	−1.0	0.009	−0.7	0.047	0.1	0.870	<0.1	0.869	−0.2	0.199

Abbreviations: BMI, body mass index; Δ, change; SAT, subcutaneous adipose tissue; VAT, visceral adipose tissue; WC, waist circumference.

a *β*-coefficients from the regression analyses are age- and sex-adjusted and represent the change in anthropometric parameters or gene expression (left column) for every 0.1 unit increase in the SAT-methylation level.

b Data derived from whole-body magnetic resonance imaging available for 49 participants.

c Average time between baseline examination (1994–1998) and the current re-examination was 14.5 years.

d Referring to *LPL* gene expression for *LPL*CG1 and -CG2, to *ADIPOQ* gene expression for *ADIPOQ*-CG1 and -CG2 and to *PPARγ* gene expression for *PPARγ*–CG1. Data were normalized to the expression of 18S rRNA and relative to the weakest expression observed in this study population.
