# Supplementary material for: Regional distribution of body fat in relation to DNA methylation within the LPL, ADIPOQ and PPARγ promoters in subcutaneous adipose tissue
Source: Nutr Diabetes. 2015 Jul 6;5(7):e168–. doi: 10.1038/nutd.2015.19 (PMC4521174; doi:10.1038/nutd.2015.19)
Supplement: Supplementary Table 1 [file nutd201519x1.doc]

**Supplementary Table 1: Reaction conditions and primer sequences of the bisulfite-PCRs.**

| DMR | Sequence forward primer | Sequence reverse primer | AT (°C) | C |
| --- | --- | --- | --- | --- |
| *ADIPOQ* | 5´-tttttttggtttaattagtttgttaaat-3´ | 5´-ttcttacaaaccacacattctaataaa-3´ | 54 | 42 |
| *LPL* | 5´-tttgttaatgttaaaatattagattgt-3´ | 5´-cttaacttaaaaaattccactcta-3´ | 54 | 40 |
| *PPARγ* | 5´-ttgatgttttgatttatgggtgtattta-3´ | 5´-aaacacaacctaaaaaacaaactacaaa-3´ | 54 | 44 |

PCRs on bisulfite-treated DNA were performed in 30 µl reaction volume in presence of 1xbuffer B, 2.5 mM MgCl_2_, 0.06 mM of each dNTP, 0.2 µM of each primer, 2.5 U HotFire DNA polymerase (Solis BioDyne, Tartu, Estonia) and 2 µl template. PCR programs started with a 15 min initial denaturation at 95° C followed by different cycle numbers of 95° C for 1 min, AT° C for 1 min, 72° C for 90 sec and a 10 min final extension step at 72° C. Abbreviations: AT, annealing temperature; C, number of cycles; DMR, differentially methylated region.
