# Supplementary material for: Regional distribution of body fat in relation to DNA methylation within the LPL, ADIPOQ and PPARγ promoters in subcutaneous adipose tissue
Source: Nutr Diabetes. 2015 Jul 6;5(7):e168–. doi: 10.1038/nutd.2015.19 (PMC4521174; doi:10.1038/nutd.2015.19)
Supplement: Supplementary Table 2 [file nutd201519x2.doc]

**Supplementary Table 2: Reaction conditions reaction conditions and primer sequences of the SIRPH analysis.**

| Gene | SNuPE primer CG1 | CG1 (hg19) | SNuPE primer CG2 | CG2 (hg19) | AT (°C) | Acetonitril Gradient | Oven Temperature (°C) |
| --- | --- | --- | --- | --- | --- | --- | --- |
| *ADIPOQ* | 5´-ggttatttatttaagt-3´ | 3: 186559147 | 5´-tcacattaaacc-3´ | 3: 186559187 | 50 | 12 min: 5.75% | 50 |
| *LPL* | 5´-taagtataagttggga-3´ | 8: 19796299 | 5´-gtattaaagtgt-3´ | 8: 19796421 | 50 | 12 min: 5.5% - 7% | 50 |
| *PPARγ* | 5´-aacaaaaaatcaatcc-3´ | 3: 12393021 | - | - | 50 | 8 min: 5.75% - 7.25% | 50 |

SNuPE reactions were performed starting with 2 min denaturation at 96° C followed by 50 cycles of 96° C for 30 s, AT °C for 30 sec, 60° C for 1 min. Products are loaded directly onto the DNASep^TM^ column (Transgenomic) and separated using a flow rate of 0.9 ml/min on a WAVE3500 system applying the respective acetonitril gradient. Abbreviations: AT, annealing temperature, SNuPE, single-nucleotide primer extension, hg19, position of the analysed CpG position according to the hg19 annotation.
