# Supplementary material for: Regional distribution of body fat in relation to DNA methylation within the LPL, ADIPOQ and PPARγ promoters in subcutaneous adipose tissue
Source: Nutr Diabetes. 2015 Jul 6;5(7):e168–. doi: 10.1038/nutd.2015.19 (PMC4521174; doi:10.1038/nutd.2015.19)
Supplement: Supplementary Table 3 [file nutd201519x3.doc]

**Supplementary Table 3:** **Comparison of methylation level of selected loci in the *ADIPOQ*- and *LPL*-promoter examined in DNA samples obtained from peripheral blood and SAT of 59 individuals participating in a sub-study nested within the EPIC-Potsdam study.**

|  | **Correlation** | |
| --- | --- | --- |
| **Locus** | **r_s_** | ***p*-value^a^** |
| *LPL*-CG1 | 0.27 | 0.041 |
| *LPL*-CG2 | 0.20 | 0.123 |
| *ADIPOQ*-CG1 | 0.17 | 0.192 |
| *ADIPOQ*-CG2 | 0.18 | 0.186 |

^a^ p-value for the rank order correlation between methylation level in SAT and in peripheral blood.

Abbreviations: SAT, subcutaneous adipose tissue
